# Mr. Bethune, on the Use of Mustard Cataplasm

**Published:** 1805-06-01

**Authors:** J. Bethune

**Affiliations:** Westerham


### *To the Editors of the Medical and Phyfical Journal.*

GENTLEMEN,

BEING a well-wisher to society, I have taken the liberty of communicating some practical remarks, which, if you think worth noticing, you will have the goodness to insert in your very valuable Journal.

As medical men are occasionally called in to relieve *immediately, if possible,* internal inflammatory affections, I have found, after bleeding and emptying the bowels, the application of a small cataplasm spread on leather, or callico, composed of flour of mustard and vinegar, applied near the part, answer a much more immediately good purpose than the emplast. cantharid. by producing in a much shorter space of time a greater degree of external inflammation, and vesication if required, without affecting the urinary passages.

In cases in which poultices are necessary, the patient too often continues longer suffering by not having them very frequently changed; perhaps from not attending to the proper time of removing them, the difficulty of procuring bread and milk to form them, or other causes, a bullock's bladder, partly filled with water sufficiently heated, tied at the top, applied next the skin and covered with flannel, answers every good purpose: it retains its heat a length of time, and can easily be changed with little trouble and expence. I am, &c.

*Westerham, March* 24, 1805. J. BETHUNE.

